# Significant shared heritability underlies suicide attempt and clinically predicted probability of attempting suicide

**DOI:** 10.1038/s41380-018-0326-8

**Published:** 2019-01-04

**Authors:** Douglas M. Ruderfer, Colin G. Walsh, Matthew W. Aguirre, Yosuke Tanigawa, Jessica D. Ribeiro, Joseph C. Franklin, Manuel A. Rivas

**Affiliations:** 1 Division of Genetic Medicine, Vanderbilt Genetics Institute, Vanderbilt University Medical Center, Nashville, TN USA; 2 Departments of Medicine, Psychiatry and Biomedical Informatics, Vanderbilt University Medical Center, Nashville, TN USA; 3 Department of Biomedical Data Science, Stanford University School of Medicine, Stanford, CA USA; 4 Department of Psychology, Florida State University, Tallahassee, FL USA

**Keywords:** Psychiatric disorders, Genetics

## Abstract

Suicide accounts for nearly 800,000 deaths per year worldwide with rates of both deaths and attempts rising. Family studies have estimated substantial heritability of suicidal behavior; however, collecting the sample sizes necessary for successful genetic studies has remained a challenge. We utilized two different approaches in independent datasets to characterize the contribution of common genetic variation to suicide attempt. The first is a patient reported suicide attempt phenotype asked as part of an online mental health survey taken by a subset of participants (*n* = 157,366) in the UK Biobank. After quality control, we leveraged a genotyped set of unrelated, white British ancestry participants including 2433 cases and 334,766 controls that included those that did not participate in the survey or were not explicitly asked about attempting suicide. The second leveraged electronic health record (EHR) data from the Vanderbilt University Medical Center (VUMC, 2.8 million patients, 3250 cases) and machine learning to derive probabilities of attempting suicide in 24,546 genotyped patients. We identified significant and comparable heritability estimates of suicide attempt from both the patient reported phenotype in the UK Biobank (h^2^_SNP_ = 0.035, *p* = 7.12 × 10^−4^) and the clinically predicted phenotype from VUMC (h^2^_SNP_ = 0.046, *p* = 1.51 × 10^−2^). A significant genetic overlap was demonstrated between the two measures of suicide attempt in these independent samples through polygenic risk score analysis (*t* = 4.02, *p* = 5.75 × 10^−5^) and genetic correlation (rg = 1.073, SE = 0.36, *p* = 0.003). Finally, we show significant but incomplete genetic correlation of suicide attempt with insomnia (rg = 0.34–0.81) as well as several psychiatric disorders (rg = 0.26–0.79). This work demonstrates the contribution of common genetic variation to suicide attempt. It points to a genetic underpinning to clinically predicted risk of attempting suicide that is similar to the genetic profile from a patient reported outcome. Lastly, it presents an approach for using EHR data and clinical prediction to generate quantitative measures from binary phenotypes that can improve power for genetic studies.

## Introduction

Suicide accounts for over 40,000 deaths a year in the United States alone and close to 800,000 deaths worldwide [1, 2]. Suicide attempt and ideation affect a much larger proportion of the population with estimates of attempts 10–25 times the number of individuals that die from suicide (1 million in the US, 20 million worldwide [3]) and a 6–14% lifetime prevalence of suicidal ideation [4]. Despite preventative public health efforts, rates of suicidal behavior are increasing in the U.S., particularly among young adults [5]. Epidemiological and family studies imply a substantial genetic component with estimates of the heritability of suicide behavior ranging from 17–55% [6–9]. However, large-scale genetic studies remain difficult due to challenges in phenotypic ascertainment and collecting large enough samples to have the power to identify replicable genetic associations or to directly estimate the proportion of heritability contributed from common genetic variation [10–13]. Thus, despite both the major public health impact and the strong evidence of heritability, the genetic architecture of suicidal behaviors remains poorly understood [14].

The emergence of large-scale, population-based samples where participants are phenotypically screened and genetically interrogated provides opportunities to study the genetics of phenotypes at scale. While the vast majority of individuals who attempt suicide have been diagnosed with a psychiatric disorder [15, 16], the outcome is not limited to any single diagnosis. Previous work has pointed to genetic factors independent of diagnosis [17, 18], which make population samples particularly valuable for the study of suicide attempt. The UK Biobank, has enrolled 500,000 individuals with extensive phenotypic and genetic data, including an online mental health assessment taken by over 157,000 participants. In this assessment, participants reported self-harm behaviors and specifically whether they have ever attempted suicide with the intent to end their lives. Among those questioned, over 3000 participants responded “yes,” providing a large set of suicide attempt cases with genetic data currently available and a corresponding set of population matched controls.

Parallel efforts have been utilizing large-scale clinical data (diagnoses, medications, procedures, utilization, demographics, etc.) from electronic health records (EHR) to identify features associated with suicide attempt and to apply predictive analytics to assess risk of future suicidal behaviors [19–21]. The most recent efforts in this domain have applied machine learning with high accuracy (c-statistics above 0.8–0.9) and precision (above 0.8) for suicide attempts [20] and death [19, 21–23]. While the goal is often to predict a binary outcome (e.g., suicide attempt or death) an important product generated from these approaches is a posterior probability associated with the likelihood of the outcome occurring (e.g., probability of attempting suicide at any point in time). These probabilities can be generated for every patient with relevant data at hospital- or system-scale, regardless of whether they have the outcome or not, and are well-suited to serve as quantitative phenotypes for genetic studies. Integrating predictive analytics of suicide attempt from EHR data and genetic data allows for an opportunity to provide meaningful quantitative phenotypes for all genotyped patients and not rely on the small subset of patients who have already engaged in suicidal behavior.

In this work, we exploit both the large-scale population genetic sample from the UK Biobank and a hospital based EHR and genetic sample from the Vanderbilt University Medical Center (VUMC) to study the genetics of patient reported suicide attempt along with clinically predicted probability of suicide attempt derived from validated algorithms of suicide risk [20]. We perform genome-wide association analyses on both samples, estimate heritability of each and calculate the genetic correlation between them and across hundreds of other traits to interrogate the genetic contribution to suicide attempt and predicted risk of attempting suicide. These analyses directly address how common genetic variation contributes to suicide attempt, whether a biological basis underlies clinical predictions of suicide attempt and whether clinical prediction can be used to increase the power of genetic studies by adding a quantitative dimension to a dichotomous phenotype. Importantly, the approach is generalizable and may be applied equally well to a wide variety of medical diagnoses or traits.

## Methods

### Genotyping and quality control of the UK Biobank sample

Genotyping and imputation procedures for the UK Biobank dataset have been previously described [24]. Briefly, two genotyping arrays, the UK Biobank Axiom Array (*n* = 438,427) and the UK BiLEVE Axiom Array (*n* = 49,950), were used to create the final genotype release of 805,426 loci for 488,377 individuals. Genotype quality control was performed before the data were released publicly, including removing participants with excess heterozygosity or missingness rate, and removing markers showing effects related to batch, plate, sex or array, or those demonstrating discordance across control replicates. Imputation was performed using a reference panel derived from the Haplotype Reference Consortium (HRC), the UK10K and 1000 Genomes datasets. Pre-phasing was leveraged to gain computational efficiency by imputing haploid genotypes for each sample. A total of 670,739 variants were used for pre-phasing and imputation if they were present on both arrays, passed genotype QC in all batches, had MAF > 0.0001, and had missingness < 5%. A total of 39,313,024 variants present in HRC were imputed.

Genome-wide association analysis was conducted using logistic regression with Plink v2.00a on the set of imputed variants from 337,199 unrelated individuals of white British ancestry based on self-reported ancestry and Bayesian outlier detection on the first 6 genomic PCs. The following covariates were used for the analysis: age, sex, the first four genetic principal components, and array, which denotes whether an individual was genotyped with the UK Biobank Axiom Array or the UK BiLEVE Axiom Array. Variants present on only one array were run without array as a covariate. Phenotypes were defined using UK Biobank Data-Field 20483 (Ever attempted suicide). Cases are “yes” responses (*n* = 2,433), and controls are either “no” responses, or any other response including those that did not take the survey (*N* = 334,766). Imputed dosages were filtered for having minor allele frequency greater than 1% and imputation INFO score > 0.3 resulting in a final set of 7,797,387 variants.

### Genotyping and quality control of the VUMC BioVU sample

The VUMC has a patient population of nearly 3 million individuals for whom clinical data are stored and managed in EHR. DNA has been collected on over 250,000 of these patients (as of February 2018), and linked to clinical data that are de-identified for use in genetic studies [25]. For this study, individuals had been previously genotyped on three different Illumina platforms and experiments were performed at different times. The samples consisted of 24,262 individuals genotyped on the Illumina MEGA^EX^ platform consisting of nearly 2 million markers, 6483 individuals genotyped on the Illumina Omni1M array covering nearly 1 million markers, and 4035 individuals were genotyped on the Illumina Human660W array covering 600,000 markers. We removed samples with greater than 2% missingness or abnormal heterozygosity (|Fhet| > 0.2). Variants were excluded if they had greater than 2% missingness or Hardy-Weinberg equilibrium *p*-value < 5 × 10^−5^. We excluded SNPs with minor allele frequency less than 2% and those not genotyped in HapMap2 which removed the largest proportion of SNPs. Each dataset had over 300,000 SNPs included for imputation (MEGA: *n* = 322,697, Omni1M: *n* = 689,485, Human 660 W: *n* = 496,629). Genotype imputation was performed using the pre-phasing/imputation stepwise approach implemented in IMPUTE4 / SHAPEIT using 1000 genomes phase I reference panel. Variants were excluded for having low imputation quality (INFO < 0.3). A set of SNPs QC-ed and pruned for linkage disequilibrium was used to calculate relatedness and principal components of ancestry. For pairs of highly related individuals (pihat > 0.2), one was randomly excluded. Ancestry components were used to define a homogenous population sample by visually determining cutoffs across each PC that removed individual samples deviating from the main group of European ancestry patients leaving a single cluster. Principal components were included as covariates in association analysis to account for residual ancestry confounding. MEGA samples were genotyped in five batches and variants were removed if having significantly differing frequencies (*p* < 5 × 10^−5^) between any batch and the rest of the sample within a homogenous set of individuals (*n* = 61,676). Individuals having been genotyped on multiple platforms were retained only in one with preference for being on the MEGA array. Samples were QC-ed, imputed and analyzed separately by array type and meta-analyzed together.

### Predicted probability of attempting suicide and feature quantification

The EHR-based phenotyping of suicide attempt and machine learning derived-phenotyping algorithm used here were adapted from a published predictive model of suicide attempt risk using clinical EHR data at VUMC [20]. Briefly, clinical data were collected from the de-identified repository known as the VUMC Synthetic Derivative (SD) [25]. Candidate charts were identified using self-injury International Classification of Diseases, version 9 (ICD-9) codes (E95x.xx) for all adults in the SD. Cases of suicide attempt were identified through multi-expert chart review on a candidate list of 5543 charts with self-injury codes to identify 3250 adults (aged 18 or older) with expert-validated evidence of self-harm with suicidal intent. Of these 3250 validated cases, 73 had genetic data and were included in analyses using their posterior probabilities of attempting suicide as with all other individuals. A cohort of 12,695 adults with a minimum of three visits to VUMC were drawn from the general population as the control comparison. Clinical data were preprocessed to support prediction/phenotyping including demographics; clinical diagnoses grouped from individual ICD-9 codes to Center for Medicare and Medicaid Servicers Hierarchical Condition Categories; medications grouped to the Anatomic Therapeutic Classification, level V; healthcare utilization including counts of inpatient, outpatient, and emergency department visits for each year of the preceding five years [20]. Missing data were rare because the variables measured as counts—diagnoses, medications and visits—were imputed to zeroes if not present. Zip code used to calculate area deprivation index was missing in 6% of charts, body mass index was missing in 9.9%, race was missing in 3.6%, and date of birth used to calculate age was missing in 0.7%. Multiple imputation was used to impute missing values in those instances [26].

In our prior work [20], random forests were shown to have superior discrimination performance in identifying suicide attempt risk compared to support vector machines and regression with or without penalization. With tuning parameters of 500 trees per forest and splits of the square root of the number of predictors at each node in the tree, the clinical phenotyping algorithm was trained via optimism adjustment with the bootstrap using 100 bootstraps [27]. The model used here differed from the published model only in that it did not censor clinical data n days (where *n* ranged from 7 to 730) preceding attempt. Therefore, discrimination performance was similar to the published models (AUC = 0.94 [0.93–0.95], sensitivity = 0.92, specificity = 0.82). The phenotyping algorithm was applied to 235,932 patients with genetic data in the biobank at VUMC (BioVU). We note demographic differences in these cohorts—the cohort used for clinical prediction compared to the genetic sample. The racial mix of the QC-ed genetic sample is entirely European while the clinical prediction sample is closer to 80% European. The mean, median age for the genetic sample was 64.7 years and 64.4 years respectively while those of the clinical sample were 47.2 years and 45.4 years respectively. Finally, the genetic sample was 50.7% women and 49.2% men while the clinical sample was 54.2% women and 45.5% men. Posterior probabilities were normalized using a rank-based inverse transformation and used as the quantitative phenotypes in genetic analyses (results remained stable when applying other normalization approaches, data not shown).

## Results

### Genome-wide association study (GWAS) of suicide attempt in UK Biobank

**Fig. 1:**
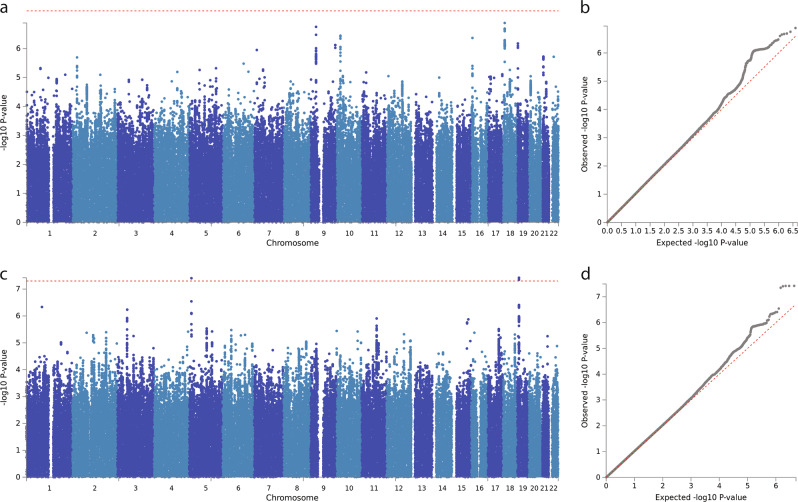
Genome-wide association results: **a** Manhattan plot for UK Biobank participants attempting suicide vs all controls, red line represents *p* = 5 × 10^−8^. **b** QQ-plot for UK Biobank suicide attempt. **c** Manhattan plot for linear regression of predicted probability of attempting suicide in BioVU, red line represents *p* = 5 × 10^−8^. d QQ-plot for predicted probability of attempting suicide in BioVU

A total of 157,366 participants provided responses to an online mental health questionnaire as a follow up to initial phenotyping in the UK Biobank sample. Of these, 6872 were asked this question from Data-Field 20483, Category: Self-harm behaviors, “Have you harmed yourself with the intention of ending your life?” Most participants were not asked this question as it required a positive response to a previous self-harm question. In total, 3563 of 6872 respondents indicated “yes”, 3089 responded “no” and 220 preferred not to answer. In an effort to maximize power and because the phenotype is rare, we included all UK Biobank participants as controls except for those responding yes to attempting suicide, this includes those that did not take the mental health assessment at all and those who preferred not to answer. After reducing our sample to a set of homogenous individuals with white British ancestry, we retained case-control data of 2433 individuals having attempted suicide and 334,766 controls across nearly 8 million variants (see Methods). No variant reached our genome-wide significance threshold of *p* < 5 × 10^−8^ (Figs. 1a, b). SNP-based heritability was estimated by LD-score regression [28] using the prevalence of suicide attempt of the participants taking the online questionnaire to convert to liability scale. We identified significant SNP-based heritability (h^2^_SNP_ = 0.035, SE = 0.01, *p* = 7.12 × 10^−4^, Table 1) in the patient reported suicide attempt phenotype.

**Table 1:** Results from heritability estimates using LD-score regression of predicted probability of attempting suicide within each genotyping array in BioVU (first three rows), all of BioVU (fourth row) and patient reported suicide attempt in UK Biobank (fifth row)

Sample	Cohort	*λ*	mean *χ*^2^	h^2^	SE	*Z*	*P*
BioVU	MEGA	1.020	1.018	0.043	0.026	1.678	9.33 × 10^−2^
	660	1.017	1.017	0.218	0.151	1.445	1.48 × 10^−1^
	Omni1M	0.999	0.996	0.148	0.109	1.359	1.74 × 10^−1^
BioVU	All	1.029	1.029	0.046	0.019	2.431	1.51 × 10^−2^
UK Biobank	All	1.038	1.038	0.035	0.010	3.385	7.12 × 10^−4^

### Polygenic risk score analysis in clinically predicted risk of attempting suicide

After QC and filtering for a homogenous set of genotyped European patients from the biobank at VUMC (BioVU), we retained 24,546 patients with high quality genotyping data across three platforms of which 73 had attempted suicide based on expert chart review. We calculated polygenic risk scores using the effect sizes calculated from the UK Biobank GWAS including all directly genotyped SNPs on each of the platforms independently. Despite small numbers, we identified a significant increase of polygenic risk among patients with a chart validated suicide attempt compared to the rest of patients in BioVU in both the single largest dataset (n = 18,128 patients, n = 40 suicide attempts, p = 0.016) and across the entire sample (n = 24,546, n = 73 suicide attempts, p = 0.033). Leveraging EHR data on almost 3 million patients at VUMC, including 3,250 with suicide attempt validated by two expert review of 5,543 charts with self-injury diagnostic codes in ICD, version 9 (co-authors JCF, JDR), we adapted a previously published machine-learning based clinical prediction model [20] (see Methods) to assign posterior probabilities of attempting suicide to all genotyped patients. We then tested the relationship between the UK Biobank based polygenic risk score and the predicted probability of attempting suicide using linear regression including four principal components and sex as covariates (including 20 principal components did not change results, data not shown). We identified a significant positive relationship between polygenic risk and predicted probability of attempting suicide in both the largest genotyped dataset (*p* = 9.96 × 10^−6^, *t*-stat = 4.42) and across all samples (*p* = 5.75 × 10^−5^, *t*-stat = 4.02), and all datasets showed positive direction (Table 2).

**Table 2:** Results from polygenic risk score analysis using UK Biobank GWAS summary statistics as discovery and testing aggregate genetic risk between BioVU patients having chart reviewed suicide attempt (left side) and quantitative probability of suicide attempt (right side) using logistic and linear regression, respectively

			Suicide attempt				Predicted risk of suicide attempt			
Sample	*N*	Validated attempt	Est	SE	T	*P*	Est	SE	T	*P*
MEGA	18,128	40	22.0	9.2	2.41	0.016	893.7	202.2	4.42	9.96 × 10^−6^
660	2,965	17	−16.2	31.4	−0.52	0.607	491.7	424.5	1.16	0.247
Omni1M	3,453	16	33.9	24.0	1.41	0.157	44.2	375.9	0.12	0.906
Total	24,546	73	18.39	8.60	2.14	0.033	659.9	164.0	4.02	5.75 × 10^−5^

*Est* regression estimate, *SE* standard error, *T* regression t-statistic, *P* p-value

### GWAS of predicted probability of attempting suicide in BioVU

We next sought to identify specific variants contributing to predicted probability of suicide attempt in BioVU. Linear regression was performed on predicted probability of suicide attempt and allelic dosage including 4 principal components of ancestry for 9 million imputed variants after filtering (see Methods). Association was performed separately for each of the genotyping platforms, and inverse weighted meta-analysis was used to combine them with Plink [29]. We identified two genomic regions containing five variants surpassing genome-wide significance (*p* < 5 × 10^−8^) on chromosomes 5 and 19 with the most significant variants being rs12972617 and rs12972618 (*p* = 3.81x10^−8^, beta = −0.063, Figs. 1c, d). However, none of these variants replicated at nominal significance (*p* < 0.05) in the UK Biobank GWAS (Supplementary Table 1). We identified significant SNP based heritability (h^2^_SNP_ = 0.046, SE = 0.019, *p* = 0.015, Table 1) at around the same level as the patient reported outcome of suicide attempt used in the UK Biobank data.

### Genetic correlation of suicide attempt and other phenotypes

**Fig. 2:**
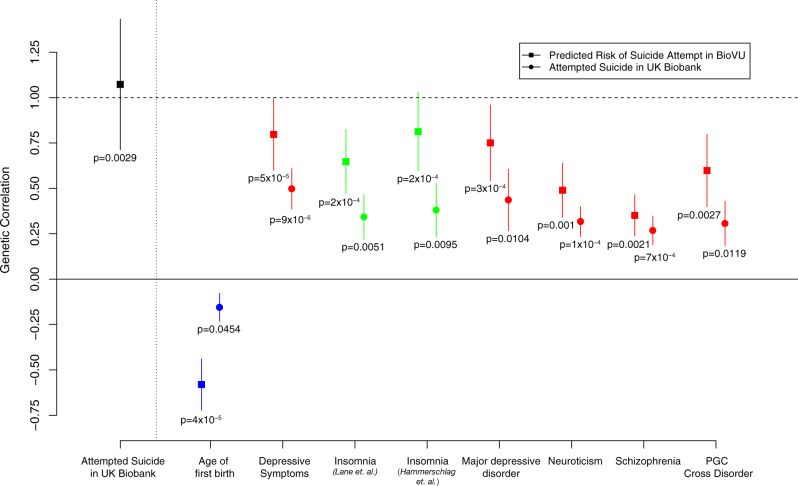
Genetic correlations and standard errors: Black point is rg between association of patient-reported suicide attempt in UK Biobank and predicted probability of attempting suicide in BioVU. Colored points represent set of phenotypes surpassing multiple test corrected significance of genetic correlation with suicide attempt after meta-analysis of UK Biobank and BioVU. Colors represent phenotype category (psychiatric = red, sleep = green, reproduction = blue). Square points are rg with predicted probability of suicide attempt in BioVU and circle points are rg with suicide attempt in UK Biobank. Complete rg results are in Supplementary Table 2

Both the GWAS of suicide attempt in UK Biobank and the GWAS of predicted probability of suicide attempt demonstrated significant heritability estimates of around 4%, and significant correlation was observed between predicted probability of suicide attempt and a polygenic risk score calculated from patient reported suicide attempt in UK Biobank. To further quantify the overlap between the genetic architecture of these two measures of the same trait we calculated genetic correlation and identified significant rg of 1.073 (SE = 0.36, *z*-score = 2.98, *p* = 0.002). We next assessed genetic correlation between GWAS summary statistics of our two suicide attempt phenotypes and 233 other GWAS traits [30]. Genetic correlations were performed for each phenotype separately and then meta-analyzed using Stouffer’s method of combining z-scores. In total, we performed 466 tests making our multiple test corrected significance threshold *p* < 1.07 × 10^−4^. Eight phenotypes surpassed this threshold in categories such as reproduction, sleep and psychiatric disorders (Fig. 2). Specifically, we identified significant positive genetic correlation of suicide attempt with depressive symptoms [31], neuroticism [31], schizophrenia [32], insomnia [33, 34], major depressive disorder [35] and a combined phenotype of five psychiatric disorders [36] as well as significant negative genetic correlation with age at first birth [37] (Supplementary Table 2). Two traits showed nominally significant genetic correlation in both phenotypes but in opposite directions including intelligence (BioVU: rg = −0.53, *p* = 3 × 10^−4^, UK Biobank: rg = 0.19, *p* = 0.044) and years of schooling [38] (BioVU: rg = −0.53, *p* = 3.3 × 10^−5^, UK Biobank: rg = 0.19, *p* = 0.007).

## Discussion

We present two large-scale genetic analyses of suicide attempt based on population samples. One from a national effort with direct assessment of suicide attempt through online questionnaire and one from a hospital system where suicide attempt risk was predicted based entirely on clinical features from EHR data. Both analyses demonstrated significant heritability estimates from common variation of around 4% and significant genetic correlation between them and with previously implicated psychiatric traits such as depressive symptoms, neuroticism, major depressive disorder and schizophrenia. These results point to a heritable component of suicide attempt and a complex underlying genetic etiology that is highly overlapping but also distinct from any single psychiatric disorder. In addition to identifying significant genetic correlations with previously implicated psychiatric traits we have identified two significant genetic correlations with non-psychiatric traits. The positive relationship between genetic risks of suicidal behavior and insomnia has been well studied with consistent evidence of the effect of disturbed sleep on suicidal behaviors including direct predictive effect of insomnia after accounting for depressive symptoms and other psychiatric traits [39, 40]. Here, we demonstrate that these two traits share a common genetic risk profile pointing to shared underlying biology consistent with previous work showing genetic correlation of insomnia and other sleep traits and psychiatric disorders [33, 34]. Additionally, we’ve identified an inverse relationship between the genetic risks of suicide attempt and age at first birth which has been shown to have a negative genetic correlation with schizophrenia [41]. Further work will be needed to dissect potential independent genetic components across these related phenotypes, however, these data point to a potential confluence of genetic risk contributing to many phenotypes that lead to increased risk of suicide attempt.

We demonstrate both a polygenic risk signature and a genetic correlation between patient-reported suicide attempt and a clinically-predicted risk of attempting suicide. These results demonstrate that clinically predicted probability of attempting suicide based only on EHR data has a genetic component that is comparable to the patient-reported phenotype. Further, we have created a quantitative value for a binary trait that significantly correlates with a quantitative measure of genetic risk. The quantitative measure enables a 39-fold increase in power (prevalence of 0.64% calculated empirically) [42], for our genetic study as evidenced by comparable estimates of heritability between the 337,000 individual UK Biobank sample with 2,433 cases and the 24,546 individual BioVU sample with 73 validated cases. The opportunities to extend this approach to additional phenotypes that are hard to ascertain or rare are extensive but, of course, require the presence of substantial clinical data and enough validated cases to successfully predict those outcomes. While we had only 24,546 samples with the genetic data from BioVU we had access to 2.8 M patients in the VUMC EHR including 3250 chart confirmed cases of suicide attempt to perform prediction and calculate posterior probabilities. Clinical data are becoming more available, more accessible, more detailed and integrated into larger systems further enabling this approach to power future genetic studies of many phenotypes.

Despite the increase in power from using the posterior probabilities, we identified only two genome-wide significant loci, both of which did not replicate in the UK Biobank sample and no genome-wide significant loci were identified in the UK Biobank sample alone. For genetic studies of suicide attempt, the samples sizes used here were large but these numbers are small when compared to the number of samples required before other psychiatric and non-psychiatric phenotypes successfully identified loci with GWAS. Based on the polygenic analysis and the significant estimates of h^2^_SNP,_ we anticipate the identification of many genome-wide significant loci of suicidal behavior as sample sizes continue to increase. The h^2^_SNP_ estimates are significant but low compared to estimates from family studies, but similar differences have been seen in several psychiatric disorders and these values reflect only contribution from common variation. Additionally, these are potentially conservative estimates due to methodological choices (e.g., LD-score regression without constraining intercept) and comparison to all samples as opposed to only healthy controls. Further, while the genetic correlation between the two suicide attempt phenotypes is high the standard error is large leaving a substantial confidence interval ranging as low as 0.71. The genetic correlations with other phenotypes show consistency across the two independent phenotypes although we note some circularity in that diagnostic codes were included in building the predictive model. We do see some differences between the two phenotypes including opposite directions of genetic correlation with intelligence and educational attainment. This difference appears to correspond with a difference in measured fluid intelligence among those that participated in the online mental health survey and looking only at those who responded to the question about suicidal behavior we saw reduced fluid intelligence among those who have attempted compared to those that responded no, similar direction to what was seen in the VUMC cohort. This represents just one example of potential differences that are likely a product of sample population or ascertainment which differ substantially between a hospital population phenotyped from clinical data and a national population sample of largely healthy individuals (between age 40 and 69) phenotyped based on a single question from an online survey. Further work and increased sample sizes will be required to determine the true genetic correlation of these phenotypes and how they differ.

Taken together, our results point to a significant genetic component of suicide attempt and a significant but incomplete genetic relationship with psychiatric and sleep traits. We demonstrate that utilizing clinical data from EHR and machine learning approaches can generate quantitative risk probabilities that share substantial genetic etiology to a more classically used patient-reported outcome. Finally, we show that these quantitative probabilities can be used to substantially improve the power of genetic studies relative to relying on the binary trait alone.

## Supplementary information

Supplementary Tables
